# Examining the Role of Components of *Slc11a1* (*Nramp1*) in the Susceptibility of New Zealand Sea Lions (*Phocarctos hookeri*) to Disease

**DOI:** 10.1371/journal.pone.0122703

**Published:** 2015-04-14

**Authors:** Amy J. Osborne, John Pearson, B. Louise Chilvers, Martin A. Kennedy, Neil J. Gemmell

**Affiliations:** 1 Department of Anatomy, University of Otago, Dunedin, New Zealand; 2 Department of Pathology, University of Otago, Christchurch, New Zealand; 3 Department of Public Health and General Practice, University of Otago, Christchurch, New Zealand; 4 Marine Species and Threats Team, Department of Conservation, Wellington, New Zealand; 5 Allan Wilson Centre for Molecular Ecology and Evolution, University of Otago, Dunedin, New Zealand; The University of Hong Kong, HONG KONG

## Abstract

The New Zealand sea lion (NZSL, *Phocarctos hookeri*) is a Threatened marine mammal with a restricted distribution and a small, declining, population size. The species is susceptible to bacterial pathogens, having suffered three mass mortality events since 1998. Understanding the genetic factors linked to this susceptibility is important in mitigating population decline. The gene solute carrier family 11 member a1 (*Slc11a1*) plays an important role in mammalian resistance or susceptibility to a wide range of bacterial pathogens. At present, *Slc11a1* has not been characterised in many taxa, and despite its known roles in mediating the effects of infectious disease agents, has not been examined as a candidate gene in susceptibility or resistance in any wild population of conservation concern. Here we examine components of *Slc11a1* in NZSLs and identify: i) a polymorphic nucleotide in the promoter region; ii) putative shared transcription factor binding motifs between canids and NZSLs; and iii) a conserved polymorphic microsatellite in the first intron of *Slc11a1*, which together suggest conservation of *Slc11a1* gene structure in otariids. At the promoter polymorphism, we demonstrate a shift away from normal allele frequency distributions and an increased likelihood of death from infectious causes with one allelic variant. While this increased likelihood is not statistically significant, lack of significance is potentially due to the complexity of genetic susceptibility to disease in wild populations. Our preliminary data highlight the potential significance of this gene in disease resistance in wild populations; further exploration of *Slc11a1* will aid the understanding of susceptibility to infection in mammalian species of conservation significance.

## Introduction

The regularity with which marine mammals are subject to epizootic episodes is well documented [1] and mass mortality events are far from rare [2]. Many pinniped species have undergone mass mortalities in recent years, often due to viral pathogens such as influenza [3], herpesvirus [4] and more commonly morbilivirus [5–8], however bacterial infection has also been seen [9]. The New Zealand sea lion (*Phocarctos hookeri*, NZSL) appears to be highly susceptible to infection by bacterial pathogens. Episodic disease events have been a frequent occurrence in the recent history of the NZSL population; three outbreaks have been documented since 1998, resulting in high levels of mortality of pups especially, but also of adults in one event [2, 10, 11]. For the approximately 40 years prior to the mass mortality events of 1997/1998 and beyond, the NZSL population level had been static (Taylor, 1971, Wilkinson et al., 2003, Wilkinson et al., 2006). While pinniped populations often recover after mass mortality events, the NZSL population has not, and is now in decline [12]. Resource competition with fisheries is a significant cause of decline of the NZSL population [13–15], but disease episodes resulting in high mortality may also be significantly impacting population growth [14]. Thus, understanding the nature of the susceptibility of the NZSL to these bacterial pathogens is important if further population decline is to be avoided.

It is suggested that novel disease episodes within a species are not usually caused by new infectious organisms, rather they are caused by ‘host shifts’, where known agents infect new hosts [16]. Therefore much can be understood by investigating genes that have known involvement in disease resistance and/or susceptibility in other mammalian species. A common point of focus is the major histocompatibility complex (MHC) and other parts of the acquired immune system (e.g.[17–28]). However, elements of innate immunity systems have been seldom explored, but are known to contribute to susceptibility or resistance to a variety of pathogens (e.g.[29]).

As a step towards addressing the absence of data on the elements of innate immunity, here we investigate the gene solute carrier family 11 member a1 (*Slc11a1*, previously known as natural resistance associated macrophage protein 1 *Nramp1*), which is involved in resistance to a broad array of bacterial pathogens [30]. While this gene has never before been studied in wildlife species of conservation concern, it is an excellent candidate through which genetic susceptibility or resistance to bacterial infection might be conferred. *Slc11a1* plays an important role in innate immunity, preventing bacterial growth in the early stages of infection [31]; it may either contribute to bactericidal activities of macrophages or be involved in more general processes of macrophage activation [32]. *Slc11a1* is expressed exclusively in macrophages and polymorphonuclear leukocytes [31, 33, 34] and encodes a membrane protein that has structural homology to transport proteins [31]. It is involved in the movement of iron ions from macrophage phagolysosomes to the cytoplasm, and because excess iron in phagolysosomes supports pathogen proliferation [35] the pump function starves the phagolysosomal compartment, and consequently the pathogen, of this essential cation [36] and prevents replication of intracellular parasites. The SLC11A1 protein has a structure and function that has been highly conserved through evolution [31, 37]. *Sllc11a1* homologs are widely identified and highly conserved from yeast to humans [38], suggesting an ancient origin of at least one billion ya [39].

*SLC11A1* is implicated as a strong candidate for human susceptibility to tuberculosis [40, 41], and has been associated with autoimmune and infectious disease in human conditions such as rheumatoid arthritis, multiple sclerosis, pulmonary tuberculosis, visceral leishmaniasis and meningococcal meningitis [30, 42]. In other species, *Slc11a1* has been shown to have a broad role in resistance to multiple bacterial pathogens. A microsatellite detected in the 3' untranslated region of *SLC11A1* is associated with resistance to brucellosis infection in cattle and water buffalo [43–47], paratuberculosis (Johne’s disease) in cattle [48] and resistance to and severity of paratuberculosis infection in sheep [49]. In mice, *Slc11a1* is associated with resistance to intracellular pathogens such as *Mycobacterium*, *Salmonella* and *Leishmania* [31, 50–53]. In dogs, polymorphisms residing in the promoter region [54, 55], microsatellite length variants within intron 1 of the gene [55], and the complete deletion of exon 11 (encoding a consensus transport motif of the protein) [55] are all associated with *Leishmania* susceptibility. Finally, in chickens, a single amino acid change was identified only in cell lines susceptible to *Salmonella enterica* [56]. Thus, data from multiple species suggests a complex and broad role for *Slc11a1* in resistance to multiple strains of bacterial pathogen and we hypothesise that this gene may be influencing NZSL resistance to novel bacterial pathogens.

Here we describe sequenced regions of the *Slc11a1* that have been previously associated with resistance to bacterial infection in dogs [54, 57]. We aimed to identify conserved elements within the gene that would be indicative of conserved function between mammalian taxa, and polymorphisms that may be linked to susceptibility and resistance to bacterial pathogens in the NZSL using a cohort of animals where cause of death was known.

## Methods

### Sampling

The cohort used here consists of 93 live NZSL pups, and a further 92 with known causes of death as determined by autopsy. Samples were collected on public land between the 2000/2001 and the 2004/2005 Austral summer breeding seasons from Sandy Bay, Enderby Island, in the Auckland Islands group (50°42′S 166°5′E). Dead pups had been assigned a known cause of death at autopsy, and here we include those that died from bacterial infection (n = 23), hookworm-related enteritis (n = 32) and ‘other’ (n = 37) where ‘other’ includes pups that died from causes other than pathogenic (trauma, malnutrition or stillbirth).

The NZSL is a protected species under the Marine Mammal Protection Act (1978), administered by the New Zealand Department of Conservation (DOC). Samples presented in this paper were collected with funding from the NZ Department of Conservation, and approvals for sample collection were obtained from the DOC Animal Ethics Committee (Approvals AEC52 1 June 2002 and AEC86, 31 December 2004).

### PCR amplification and sequencing

DNA was extracted as described in Osborne *et al*. 2013 [17]. Part of the promoter region of *Slc11a1* (377bp) was amplified using oligonucleotides previously designed for application to the dog genome [57]: NRAMP1-F 5’-CCTCTCAGCTAGTCTGAGCC—3’ and NRAMP1-R 5’—CAGCTGATCTCAGCTGTCCTC—3’. Amplification of the partial promoter region was achieved by polymerase chain reaction (PCR) of genomic DNA in 10μL reaction volumes containing *ca*. 50ng template DNA, 20mM Tris-HCl, 50mM KCl, 5nmol each dATP, dTTT, dGTP and dCTP, 1pmol each primer, 2mM MgCl_2_, 6% DMSO and 0.1 unit *Taq-Ti* DNA polymerase (Fisher Biotec, Australia). Touch-down thermal cycling parameters [58] were as follows: initial denaturation at 94^°^C for 5 minutes, followed by 10 cycles of 94^°^C for 20 seconds, 65^°^C for 20 seconds, and 72^°^C for 30 seconds. Next, 40 cycles of 94^°^C for 20 seconds, annealing temperature (Tx) for 20 seconds and 72C for 30 seconds were performed, where Tx decreased by 0.5^°^C per cycle, beginning at 65^°^C. This was finalised by an extension step of 72^°^C for 5 minutes. PCR products were sequenced using an ABI 3730xl DNA Analyser (Applied Biosystems, Carlsbad, CA, USA) using the Genetic Analysis Service at the University of Otago and analysed with the program Geneious [59]. NZSL-specific primers for *Slc11a1* were designed with the program Primer3 [60]: NZSL_F 5’—GAAGAACCAAGTTCAGAGAAAGG-3’ and NZSL_R 5’- TCTGGCGGAAGAGTCTTGT-3’, using DNA sequences that were successfully obtained from PCR on NZSL DNA with canine primers, and PCR amplification was carried out as described above.

### Intron 1 microsatellite genotyping

The intron 1 microsatellite was amplified using primers designed to the dog genome [55]: NRMICRI1-F 5’—TGTAAAACGACGGCCAGTGAGTCTGCTTGAGATTCTCTC—3’, NRMICRI1-R 5’—TATCACCTCCACCCTTCAAAC—3’. The forward primer was fluorescently labelled with a 5’ M13 tag (underlined above) for subsequent genotyping [61], for which reaction conditions were *ca*. 50ng template DNA, 20mM Tris-HCl, 50mM KCl, 5nmol each dATP, dTTT, dGTP and dCTP, 1pmol M13 primer, 1pmol reverse primer, 0.25pmol forward primer, 2mM MgCl_2_, 6% DMSO, 75mM TMAC and 0.1 unit *Taq-Ti* DNA polymerase (Fisher Biotec, Wembley, WA, Australia). Thermal cycling parameters were as follows: initial denaturation 94°C for 5mins; 35 cycles of: 94°C for 30 seconds, 45°C for 30 seconds, 72°C for 45 seconds; 8 cycles of: 94°C for 30 seconds, 53°C for 30 seconds, 72°C for 45 seconds; final extension 72°C for 10 mins. PCR products were genotyped using an ABI 3730xl DNA Analyser and analysed with the program GeneMapper (both Applied Biosystems, Carlsbad, CA, USA). For microsatellite analyses only, the number of individuals analysed were: n = 18 (bacteria), 30 (enteritis), 34 (other). These differences are due to differing PCR success between the *Slc11a1* partial promoter sequence and the microsatellite.

The products amplified from oligonucleotide primers for the intron 1 microsatellite were sequenced in dead NZSL pups by Sanger sequencing methods [62] to ensure the correct region had been isolated. Sequencing through repetitive regions often leads to error and can mean the sequence is unreadable beyond the repeat region. Therefore, while this method is not ideal since only the flanking regions are amplified, it is sufficient for the purpose of identifying the amplified region. The sequence obtained for the forward strand aligned to canine *Slc11a1* sequence upstream of the repeat region, and the reverse strand aligned (in reverse complement) downstream of the repeat region, indicating that the correct region of NZSL DNA was being amplified.

### Transcription factor binding prediction

The program MatInspector [63] was used to identify putative transcription factor binding sites, and from these we determined what effect the base substitution had on the integrity of predicted transcription factor binding sites.

### Comparative analysis by sequence alignment

The integrity of the region of the NZSL promoter amplified here was investigated in other mammals through multiple sequence alignment of cattle (*Bos taurus*, AY438096), sheep (*Ovis aries*, AF128882), pig (*Sus scrofa*, EU135795) human (*Homo sapiens*, NG_012128) and dog (*Canis familiaris*, AF091049) *Slc11a1* sequences. Mammalian sequences were aligned to the NZSL promoter region using the default alignment method in Geneious [59]. We also looked for evidence of intraspecific polymorphism in several mammalian species, for which adequate data were available, using the dbSNP database for Short Genetic Variations (http://www.ncbi.nlm.nih.gov/SNP/), with search terms limited to exclude ‘homo’ (*Homo sapiens*) sequence submissions. Retrieved sequences were aligned using MAFFT (Multiple Alignment using Fast Fourier Transform, http://www.ebi.ac.uk/Tools/msa/mafft/help/).

### Statistical analyses

The statistics package R [64] was used to explore the association between i) the promoter polymorphism, and ii) length variation in the intron 1 microsatellite with cause of death in the NZSL pup population by use of Chi-squared tests and Fisher’s Exact Test for count data using the R package *EpiTools* [65]. Additionally, *EpiTools* was used to calculate whether or not genotypes were in Hardy Weinberg equilibrium. Where appropriate, post-hoc power analyses were undertaken in R using the program GPOWER [66]. GenePop version 4.0.10 [67] was used to investigate whether or not any linkage disequilibrium was present between the promoter polymorphism and alleles of the microsatellite. Phase v2.1 [68] was used to reconstruct haplotypes including both the promoter polymorphism and the microsatellite.

## Results

### Promoter sequence

Initial PCR of NZSL DNA with primers designed to the dog genome was inefficient and amplified only ~80% of individuals, likely due to lack of full complementarity between primers and the target sequence due to the presence of primer site polymorphisms in some individuals. New primers (NZSL_F and NZSL_R) designed to NZSL DNA from those individuals that were successfully amplified and sequenced with the canine primers resulted in consistent amplification of 377bp of promoter sequence after trimming (S1A Fig). This sequence shared 85% identity with the canine *Slc11a1* sequence (accession number AF091049). A single polymorphic site was detected in this region, a guanine (G) to adenine (A) substitution at residue number 317 of the 377bp amplified here (S1A Fig). Both heterozygotes and homozygotes were detected at this site (S1 Fig) and genotype and allele frequencies are reported in Table 1.

**Table 1:** Genotype and allele frequencies of *Slc11a1* promoter polymorphism in NZSL pups.

		Bacteria n = 23	Enteritis n = 32	Other n = 37	Live n = 93
**Genotype**	AA	17 (4)	19 (6)	24 (9)	19 (18)
	AG	35 (8)	38 (12)	49 (18)	42 (39)
	GG	48 (11)	44 (14)	27 (10)	39 (36)
**Allele**	A	35 (8)	38 (12)	49 (18)	40 (37.5)
	G	65 (15)	63 (20)	51 (19)	60 (55.5)

Genotype and allele frequencies of *Slc11a1* promoter polymorphism in NZSL pups. Percentage frequencies are reported, followed by actual counts in parentheses. NZSLs are grouped according to cause of death (bacteria, enteritis, other) or live.

### Transcription factor binding motifs

The promoter region amplified here showed two putative transcription factor binding motifs of interest: one NF-I site (transforming growth factor-inducible NF-I transcription factor, recognition sequence 5’-YGGMN_5-6_GCCAA-3’) and seven putative IFN-γ (interferon gamma, recognition sequence 5’-CWKKANNY-3’) sites (S2 Fig). These transcription factor binding motifs have previously been identified in the promoter region of the canine *Slc11a1* gene [55]. A putative NF-I recognition sequence overlapped with the identified polymorphism in the NZSL promoter sequence.

Other predicted binding sites that occur in the NZSL promoter sequence are: AP1 (activator protein-1, 5’-TGASTMA-3’, one site), AP2 (activator protein-2, 5’-CCCMNSSS-3’, one site), GM-CSF (granulocyte macrophage colony-stimulating factor, 5’-CATTW-3’, two sites).

### Comparative analysis by sequence alignment

A region of canine *SLC11A1* identified as highly variable and associated with disease susceptibility [55, 57] was interrupted in the promoter of NZSL *Slc11a1* (S3 Fig). Two notable features from the alignment of NZSL with other mammals were i) the absence in non-human mammals of a repeat region present in humans, and ii) the highly variable region in dogs remains largely intact in all other species, but is interrupted in the NZSL (S4 Fig). A database search of dbSNP retrieved 390 *Slc11a1* sequence submissions. Of these 390, 180 spanned the polymorphic site identified in the NZSL promoter region and included data from *Bos taurus* (cow, n = 1 sequence), *Canis familiaris* (dog, n = 8 sequences), *Mus musculus* (mouse, n = 136 sequences), *Ovis aries* (sheep, n = 1 sequence), *Pan troglodytes* (chimpanzee, n = 9 sequences), *Pongo abelii* (orangutan, n = 6 sequences) and *Rattus norvegicus* (rat, n = 19 sequences). Based on MAFFT alignment, intraspecific polymorphism was detected at the NZSL polymorphic site in all species above, but for those species where only one sequence was retrieved (cow and sheep).

### Statistical analysis of promoter polymorphism

In the first instance, genotypes at the promoter polymorphism were identified through Sanger sequencing methods [62], and each animal was categorised according to status (live or dead) and cause of death (bacteria, enteritis, other, where ‘other’ is defined as death from trauma, malnutrition or stillbirth, Table 1).

No differences in genotype frequency distribution were observed between status groups, as determined by Fisher’s Exact Tests, Table 2 (Fisher’s Exact Test number 1, AG and GG versus AA genotype: AG, OR 1.01, 95% CI 0.19, 4.91, p-value 1; GG, OR 0.86, 95% CI 0.17, 3.92, Fisher’s p value 0.59). Similarly, no statistically significant differences were found between status groups when each allele (G, A) was considered individually (Fisher’s Exact Test number 2, A versus G allele: G, OR 0.89, 95% CI 0.39, 1.97, p-value 0.42). When all dead pups, regardless of cause, were combined and compared to live pups, no difference in allele frequency was observed (Fisher’s Exact Test number 3, A versus G allele: G, OR 1.04, 95% CI 0.69, 1.58, p-value 0.92).

**Table 2:** Fisher’s Exact Tests of NZSL *Slc11a1* promoter polymorphism by disease status.

**1) BACTERIAvs.ENTERITISvs.OTHERvs.LIVE**				** **
**Genotype**	**OR**	**95%CI**	**Fisher's P**	**ChiSq P**
**AA**	1	NA	NA	NA
**AG**	1.01	0.19, 4.91	1	0.99
**GG**	0.86	0.17, 3.92	0.59	0.57
**2) Allele**	**OR**	**95%CI**	**Fisher's P**	**ChiSq P**
**A**	1	NA	NA	NA
**G**	0.89	0.39, 1.97	0.42	0.41
**3) LIVEvsDEAD**				
**Allele**	**OR**	**95%CI**	**Fisher's P**	**ChiSq P**
**A**	1	NA	NA	NA
**G**	1.04	0.69, 1.58	0.92	0.85
**4) (BACTERIA+ENTERITIS)vsOTHER**				
**Genotype**	**OR**	**95%CI**	**Fisher's P**	**ChiSq P**
**AA**	1	NA	NA	NA
**AG**	0.99	0.32, 3.10	1	1
**GG**	0.45	0.13, 1.47	0.23	0.17
**5) Allele**	**OR**	**95%CI**	**Fisher's P**	**ChiSq P**
**A**	1	NA	NA	NA
**G**	0.61	0.33, 1.10	0.13	0.09

Fisher’s Exact test of genotypes (AA, AG, GG) and alleles (A, G) and disease status. 1) Genotypes of all disease states (bacteria versus enteritis versus other versus live); 2) Alleles of all disease states, as in 1; 3) Alleles of live animals versus dead animals (all causes of death combined); 4) Genotypes of infected animals (those that died of bacteria or enteritis) versus uninfected (those that died of other causes); 5) Alleles of infected versus uninfected animals.

**Fig 1:**
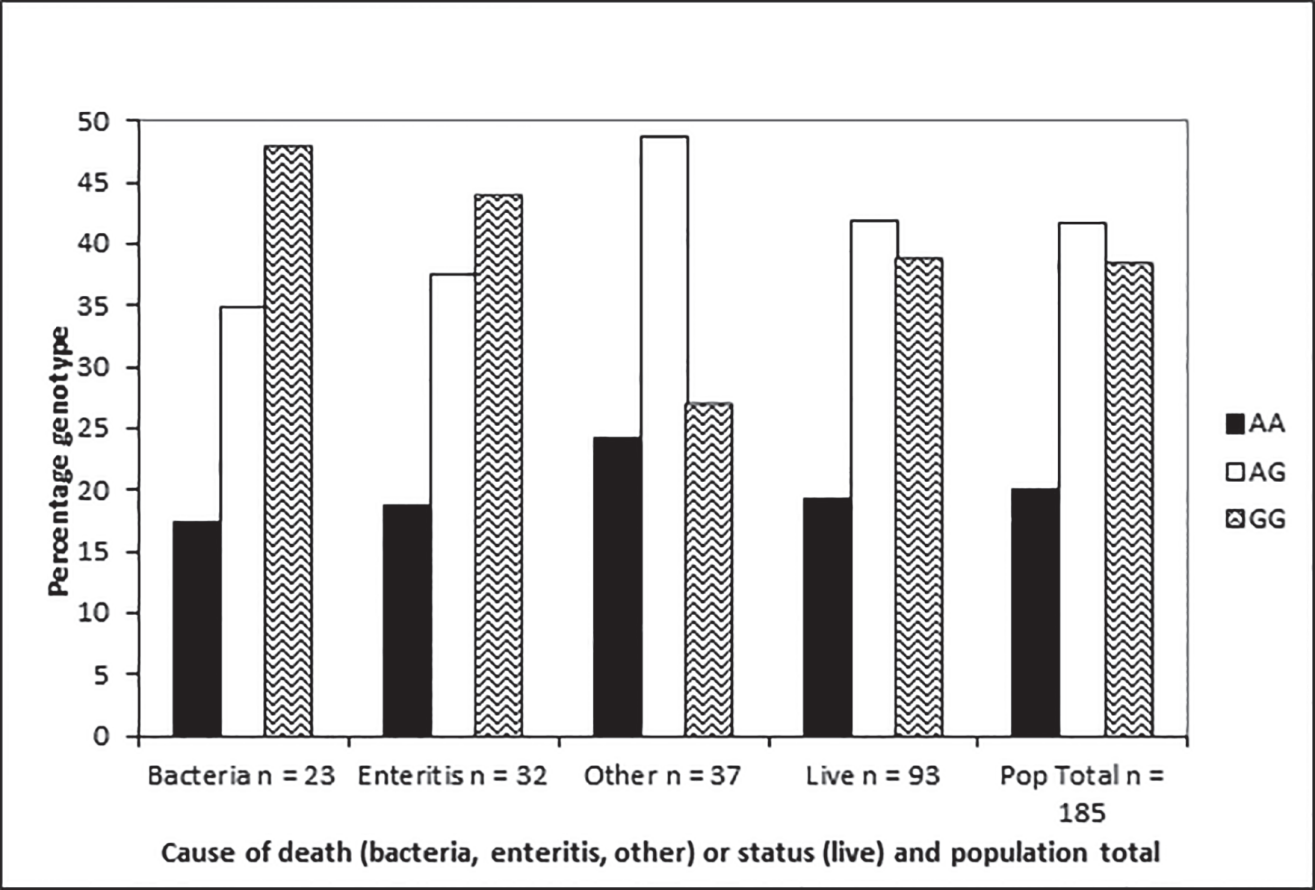
Genotype frequencies of NZSL *Slc11a1* promoter polymorphism. Genotype frequencies of *Slc11a1* promoter polymorphism (as a percentage) by class, n = 185. NZSL classes are animals with known causes of death (bacterial, enteritis, ‘other’) and live pups

Genotype frequencies for animals dying of bacteria and enteritis appear to be inconsistent with neutrality when displayed on a bar plot (Fig 1), and display a shift towards the GG homozygote state, compared to pups dying of other causes, which have a more ‘classic’ appearance of alleles at Hardy Weinberg equilibrium (Fig 1). Despite the bias towards the G allele in pups dying of infectious causes (bacteria, enteritis), genotypes are statistically considered to be in Hardy-Weinberg equilibrium (Table 3).

**Table 3:** Hardy Weinberg equilibrium calculations of NZSL *Slc11a1* promoter polymorphism.

**Hardy Weinberg**	**OTHERvs.BACT**	**OTHERvs.ENT**	**OTHERvs.LIVE**
**ChiSq**	0.59	0.72	1.16
**p-value**	0.44	0.39	0.28

Hardy Weinberg equilibrium calculations on NZSL pups. Those that died of ‘other’ causes were compared to those that had died of either bacterial infection, enteritis, or those that were live at the time of sampling.

**Fig 2:**
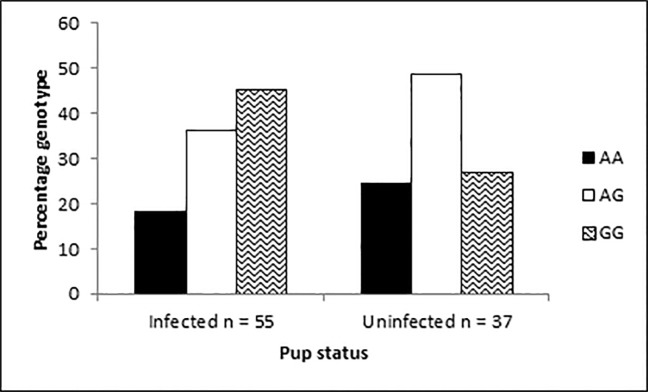
*Slc11a1* genotype frequencies of infected vs. uninfected NZSLs. Percentage genotype frequencies of *Slc11a1* promoter polymorphism when dead NZSL pups were classified as infected (those with bacterial infection or enteritis) vs. uninfected (those with ‘other’ causes of death)

Data from animals dying of infection (bacteria, enteritis) were combined and compared with those that died of other causes (essentially an analysis of ‘infected’ versus ‘uninfected’, Table 2 and Table 4), and a shift towards the G allele in infected individuals was observed (Fig 2). Animals possessing the GG genotype were twice as likely to be infected than animals possessing the AA genotype (Table 2, Fisher’s Exact Test number 4, odds ratio 0.45, 95%CI: 0.13,1.47, p-value = 0.23). Likewise, animals possessing the G allele were twice as likely to be infected than animals possessing the A allele (Table 2, Fisher’s Exact Test number 5, odds ratio 0.61, 95%CI: 0.33, 1.10, p-value = 0.13). Since these odds ratios showed p > 0.05, the β value for post-hoc statistical power of analysis was calculated, which was 0.38 [66]. The observed β value is below the threshold required to infer accurate conclusions about significance [69], and is a consequence of the number of individuals used in this study.

**Table 4:** NZSL *Slc11a1* promoter polymorphism genotype counts.

**Genotype**	**Infected**	**Uninfected**
**AA**	10	9
**AG**	20	18
**GG**	25	10

Genotype counts of dead pups. Those dying of bacteria and enteritis are here classed as 'infected' and pups dying of other causes classed as ‘uninfected’.

### Intron 1 microsatellite polymorphism

Amplified alleles were assigned numerical identifiers based on their observed fragment size. Of the alleles amplified, two (165bp and 167bp) were common; allele 165 was more frequent than allele 167 (84.15% and 11.59% respectively, Table 5). No significant difference in microsatellite allele frequency between status groups was detected (χ^2^ = 0.86; p = 0.65). Expected heterozygosity at this locus for all dead pups is 0.28 versus an observed heterozygosity of 0.22 indicating that the microsatellite, while polymorphic, shows little variation. We find no evidence for linkage disequilibrium between the intron 1 microsatellite and the promoter polymorphism for any status group, or across the population (bacteria p = 0.89; enteritis p = 0.06; other p = 0.73; total p = 0.37).

**Table 5:** *Slc11a1* intron 1 microsatellite allele frequency.

**Allele**	**Bacteria**	**Enteritis**	**Other**	**Population Total**
**161**		1.67 (1)		0.61 (1)
**165**	86.11 (31)	80.00 (48)	86.76 (59)	84.15 (138)
**167**	13.89 (5)	13.33 (8)	8.82 (6)	11.59 (19)
**168**		1.67 (1)		0.61 (1)
**169**			1.47 (1)	0.61 (1)
**171**			2.94 (2)	1.22 (2)
**173**		3.33 (2)		1.22 (2)

Intron 1 microsatellite allele frequencies (%) according to cause of death (counts in parentheses).

### Haplotype analyses

The program Phase v2.1 [68] was used to determine distinct haplotypes (the combination of intron 1 microsatellite and promoter polymorphism) for each individual with a known cause of death, to explore if certain haplotypes/combinations were more frequent in bacterial infection, enteritis infection, or other causes of death (Table 6). Haplotypes containing microsatellite alleles other than 165 and 167 of the intron 1 microsatellite appear relatively infrequently.

**Table 6:** Reconstructed haplotypes of NZSL *Slc11a1* promoter polymorphism and intron 1 microsatellite.

**Haplotype number**	**Alleles**		**Bacteria**	**Enteritis**	**Other**
**H1**	161	A	-	1	-
**H2**	165	A	11	12	25
**H3**	165	G	20	36	34
**H4**	167	A	3	6	4
**H5**	167	G	2	2	2
**H6**	168	A	-	1	-
**H7**	169	A	-	-	1
**H8**	171	A	-	-	2
**H9**	173	A	-	2	-
**Total**			36	60	68

Haplotype counts of NZSL *Slc11a1*, by cause of death. The most common haplotypes were chosen and used for downstream analyses, those being H2, H3 H4 and H5. Numbers of successful microsatellite amplifications differ slightly from *Slc11a1* sequence amplification due to PCR success.

As a consequence of low frequencies for some of the haplotypes, only haplotypes H2, H3, H4 and H5 are considered in further analyses. A bar plot of haplotype frequencies, grouped by cause of death, shows that animals dying of ‘other’ causes have a haplotype distribution of the most common haplotypes, H2 and H3, that is more reflective of neutrality than those dying of bacteria or enteritis (S5 Fig); animals dying of bacteria or enteritis show relatively high incidence of haplotype H3. Haplotype H3 is composed of the G variant of the promoter polymorphism and microsatellite allele 165. Analysis with Pearson’s Chi-squared test found no significant differences in haplotype frequencies between the three causes of death (χ^2^ = 4.78, p = 0.57). Data from infected pups were grouped (where ‘infected’ is defined as those pups dying of both bacteria and enteritis) and compared to data from pups dying from ‘other’ causes. Odds ratios as calculated by Fisher’s Exact Test showed that animals possessing haplotype H3 were twice as likely to be ‘infected’ than animals possessing haplotype H2 (OR 0.56, 95% CI: 0.26, 1.20, p-value = 0.148). However, because both haplotypes contain the same microsatellite allele, the likelihood is that this pattern is driven by the promoter polymorphism frequency differences. We note here that while the odds ratios are of interest, they were not statistically significant.

## Discussion

Here we have demonstrated conservation of gene structural elements between canids and otariids, implying potential conserved function across mammalian taxa. We have identified one polymorphism in 377bp of sequence obtained from the promoter region of the New Zealand sea lion *Slc11a1* gene. The polymorphic site lies in a putative transcription factor binding site, which was identified using canid transcription factor recognition sequences. A greater number of pups dying from infectious causes possess the G variant of this polymorphism, and while this pattern is not statistically significant, we have shown that pups with the G variant are twice as likely to be dead from infectious causes, rather than from ‘other’ causes (trauma, malnutrition, stillbirth). We provide evidence of intraspecific polymorphism in other mammalian species using the data currently available. We demonstrate conservation of a polymorphic microsatellite between canids and otariids; however there was no significant difference in frequency of the two most common microsatellite alleles between causes of death, and the locus was determined to be in Hardy Weinberg equilibrium.

Previous work with dogs has shown that haplotypes constructed of combinations of polymorphic loci analysed together (e.g. the intron 1 microsatellite and polymorphisms in G-rich regions on the promoter [57]), show associations with bacterial disease susceptibility. However, upon reconstruction, no significant difference in haplotype frequencies between different causes of death was seen, and odds ratios of association between haplotypes in infected vs. uninfected individuals were driven by the promoter polymorphism rather than the microsatellite allele. Therefore, in contrast to Sanchez-Robert *et al*. (2005), these genomic features do not appear to be interacting to produce susceptible or resistant haplotypes in the NZSL. We note that the G variant leads to a two-fold increase in likelihood that an animal is infected. This increased likelihood suggests that the polymorphism may have a role in susceptibility to infectious agents; however post-hoc power analysis of this comparison suggests that the study lacks sufficient power to test this association rigorously. A larger study including more animals with known causes of death would be required to effectively test for an association in this species.

Transcription factor binding sites identified in promoter regions, by bioinformatic analysis, can provide some clues to the function of the genes downstream of those promoters. *Slc11a1* is expressed in phagocytic cells [31, 37] and contains binding sites for interferon gamma (IFN-γ), which is involved in macrophage activation [70]. Putative IFN-γ binding sites were detected multiple times in the NZSL *Slc11a1* promoter region, consistent with the observation that IFN-γ increases the induction of S*lc11a1* gene expression in mice [33]. Indeed, the NZSL *Slc11a1* promoter region has many putative regulatory motifs which are conserved between the canid and NZSL genomes [55], consistent with the previously described role of *Slc11a1* in macrophage activation in response to IFN-γ stimulation [71].

The binding motif for nuclear factor I (NF-I), which is involved in the differentiation between erythrocytes and granulocytes [72], brain development [73] and leukaemia [74], is predicted to span the genomic region containing the promoter polymorphism in NZSLs. This raises the possibility that the polymorphism could alter the binding efficacy of this transcription factor, which might impact gene expression. The single nucleotide polymorphism (SNP) in the NZSL promoter is in a region of the transcription factor recognition sequence which has an ambiguous base; at this particular nucleotide position, either the A or the G variant would complete the sequence needed for recognition by NF-I, if the recognition sequences are functionally conserved between the NZSL and dogs. However, since transcription factor binding regions can vary in binding abilities based on changes to the target sequence [75], it is possible that the polymorphism detected here might affect the binding kinetics and efficacy of the transcription factor to the target motif in the NZSL promoter region, which could result in changes in *Slc11a1* gene expression. However, it is difficult to predict whether this SNP is affecting transcription factor binding, and confirming this would require direct biochemical analysis. We have previously shown that a high degree of homology exists between the canid and NZSL genomes [76], and it is encouraging to observe predicted shared regulatory motifs, inferring shared function and broader sequence similarity [77]. However, the distribution and nature of transcription factors in NZSL tissues is currently unknown. Although we have demonstrated conservation of regulatory sites, it is possible that these identified sites are no longer functional due to potential divergence of binding motif sequences between the canid and NZSL genomes. If the binding motifs have diverged, it could mean that different motifs are responsible for the regulation of *Slc11a1* expression in the NZSL [78, 79]. Thus, at the present time there is little possibility of predicting how changes in transcription factor binding motifs in the NZSL genome are likely to affect the binding of specific transcription factors, and the consequences for gene expression [80].

The NZSL displays a polymorphic site in a region of guanine nucleotide repeats in the promoter region, and is reflective of an identified variable region in dogs, where polymorphisms in G-rich regions have been associated with disease susceptibility [55, 57]. Polymorphisms in the G-rich regions of the *Slc11a1* promoter could have an effect on its expression [57], because differing lengths of G nucleotides in promoter regions can alter promoter activity in species as diverse as bacteria [81] and humans [82]. It is possible that interruptions of the G-rich regions of NZSL *Slc11a1* promoter region could be altering promoter activity and therefore also gene function. There is evidence of intraspecific polymorphism in published *Slc11a1* nucleotide sequences, which could mean that the NZSL polymorphic nucleotide is a commonly variable one. While the majority of the information on intraspecific polymorphism is limited to rodents, we find evidence that the polymorphic site is commonly variable in dogs as well as NZSLs (although all four nucleotides are present, in contrast to just two nucleotides found in the NZSL). The detection of intraspecific polymorphism in two related species that share a large amount of homology suggests a functional role for the nucleotide, however further work, such as *in vitro* reporter gene assays, is needed to investigate this fully.

Microsatellites within genes often affect their function [83, 84] and this has previously been demonstrated at a polymorphic microsatellite in the promoter region of human *SLC11A1* [85]. While substantial proportions of microsatellites are conserved across broad evolutionary timescales [86], normally only 40–50% of microsatellites are expected to be conserved cross-species, and approximately only half of these are expected to be polymorphic [87]. The demonstrated amplification of a polymorphic microsatellite locus here goes some way to establishing the conservation of the structure and potentially the function of this gene in the NZSL.

While distinct allele frequency differences at the promoter polymorphism were observed in relation to NZSL pup mortality and infection status, and were accompanied by a strong likelihood of infection with the G variant, these differences were not statistically significant, leading one to question the potential cause of non-significance. The NZSL is an observed population only, meaning that any variable factors can not be controlled. This is in contrast to domesticated agricultural populations where there is more control over environmental variables, and higher degrees of genetic homogeneity with populations. Higher homogeneity can lessen the variability in the phenotypes observed within the population and may strengthen the ability to detect associations between genotype and phenotype within and between populations. Environmental variability within the NZSL population is much greater than for most agricultural, laboratory and captive populations; therefore a much larger number of individuals is likely to be required to explore genetic associations with the polygenic traits likely to underlie fitness. It is likely that the factors above account for the higher p-values in detected here, despite strong odds ratios.

Thus, while a larger sample of individuals with assigned cause of death would be required to investigate more rigorously whether *Slc11a1* affects disease resistance in this species, our preliminary data, encompassing the conservation of *Slc11a1* cross-species, and the likelihoods identified here, illustrate the promising nature of this gene as a candidate for disease resistance and/or susceptibility in this and other mammalian species. We suggest that its investigation should be broadened to include wild populations and those of conservation significance.

## Supporting Information

S1 Fig S1A Fig: NZSL SLC11A1 promoter region sequence. 377bp of NZSL SLC11A1 promoter region sequence. The polymorphic site is underlined. S1B Fig: NZSL SLC11A1 promoter region sequencing trace. SLC11A1 promoter region polymorphism with variable site highlighted. Each trace represents one of three observed genotype groups in the NZSL.
(DOCX)

S2 Fig Transcription factor binding motifs. Putative transcription factor binding site motifs (AP1, NF-I, IFN-γ, GMCSF) for NZSL SLC11A1 promoter sequence, with SNP (A—G) indicated in gold. Figure output from Geneious with annotations manually added.
(DOCX)

S3 Fig Alignment of canine and NZSL partial promoter region. NZSL sequence for SLC11A1 partial promoter region aligned against canine SLC11A1 promoter region. Green bar represents sequence similarity with white gaps showing divergence. Green arrows indicate where NZSL primers bind, with missing NZSL sequence at these sites due to trimming of the sequence. Red arrow indicates the transcription start site in canine sequence. Pale arrow shows the G-rich stretch in dogs associated with susceptibility to infection. This region is interrupted in the NZSL.
(DOCX)

S4 Fig Mammalian multiple sequence alignment. Multiple sequence alignment of SLC11A1 sequences from cattle, sheep, pig, human, dog and NZSL. Variable region in canine sequence is shown shaded in blue and promoter SNP is shaded in gold in all species.
(DOCX)

S5 Fig Haplotype frequencies. Haplotype frequencies (%) of combined promoter polymorphism and microsatellite variant, grouped by cause of death. Haplotype (H) numbers refer to haplotype numbers indicated in Table 5.
(DOCX)
