# Supplementary material for: Examining the Role of Components of Slc11a1 (Nramp1) in the Susceptibility of New Zealand Sea Lions (Phocarctos hookeri) to Disease
Source: PLoS One. 2015 Apr 14;10(4):e0122703. doi: 10.1371/journal.pone.0122703 (PMC4397024; doi:10.1371/journal.pone.0122703)
Supplement: S1 Fig — 377bp of NZSL SLC11A1 promoter region sequence. The polymorphic site is underlined. S1B Fig: NZSL SLC11A1 promoter region sequencing trace. SLC11A1 promoter region polymorphism with variable site highlighted. Each trace represents one of three observed genotype groups in the NZSL. (DOCX) [file pone.0122703.s001.docx]

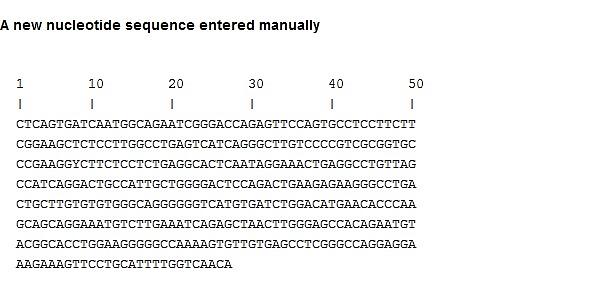


**S1a Fig. NZSL SLC11A1 promoter region sequence:**

377bp of NZSL SLC11A1 promoter region sequence. The polymorphic site is underlined.


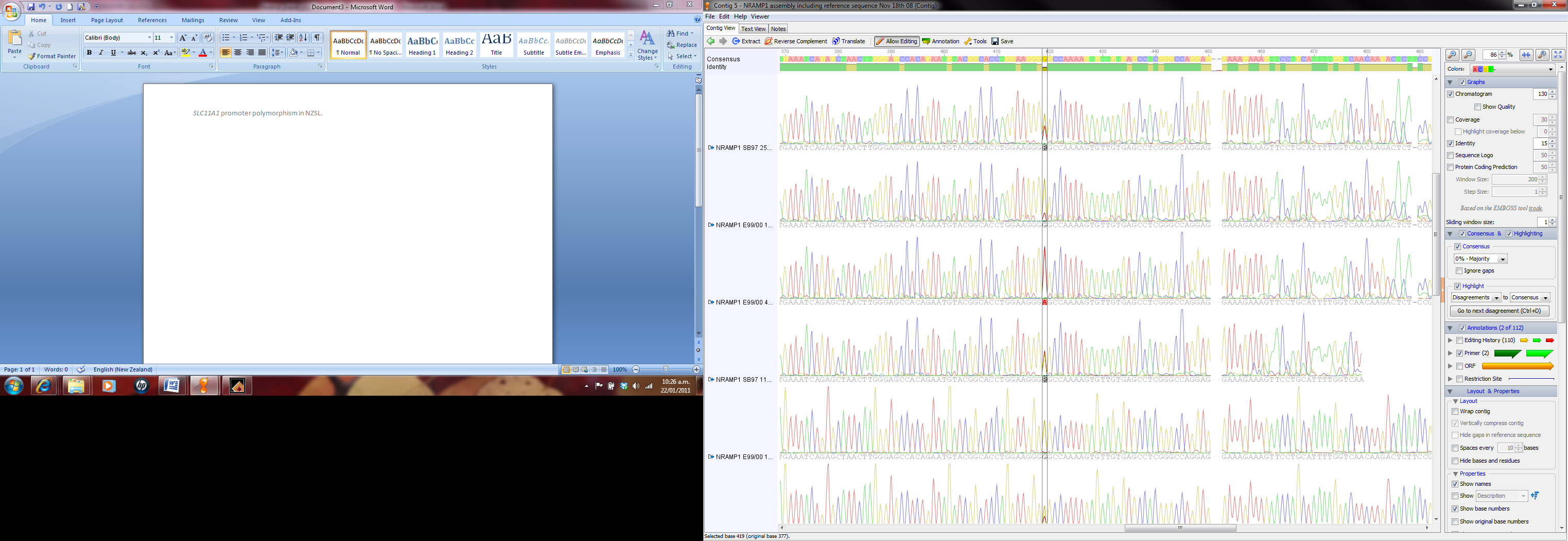


**S1b Fig.** **NZSL SLC11A1 promoter region sequencing trace**.

SLC11A1 promoter region polymorphism with variable site highlighted. Each trace represents one of three observed genotype groups in the NZSL
