# Supplementary material for: Examining the Role of Components of Slc11a1 (Nramp1) in the Susceptibility of New Zealand Sea Lions (Phocarctos hookeri) to Disease
Source: PLoS One. 2015 Apr 14;10(4):e0122703. doi: 10.1371/journal.pone.0122703 (PMC4397024; doi:10.1371/journal.pone.0122703)
Supplement: S2 Fig — Putative transcription factor binding site motifs (AP1, NF-I, IFN-γ, GMCSF) for NZSL SLC11A1 promoter sequence, with SNP (A—G) indicated in gold. Figure output from Geneious with annotations manually added. (DOCX) [file pone.0122703.s002.docx]

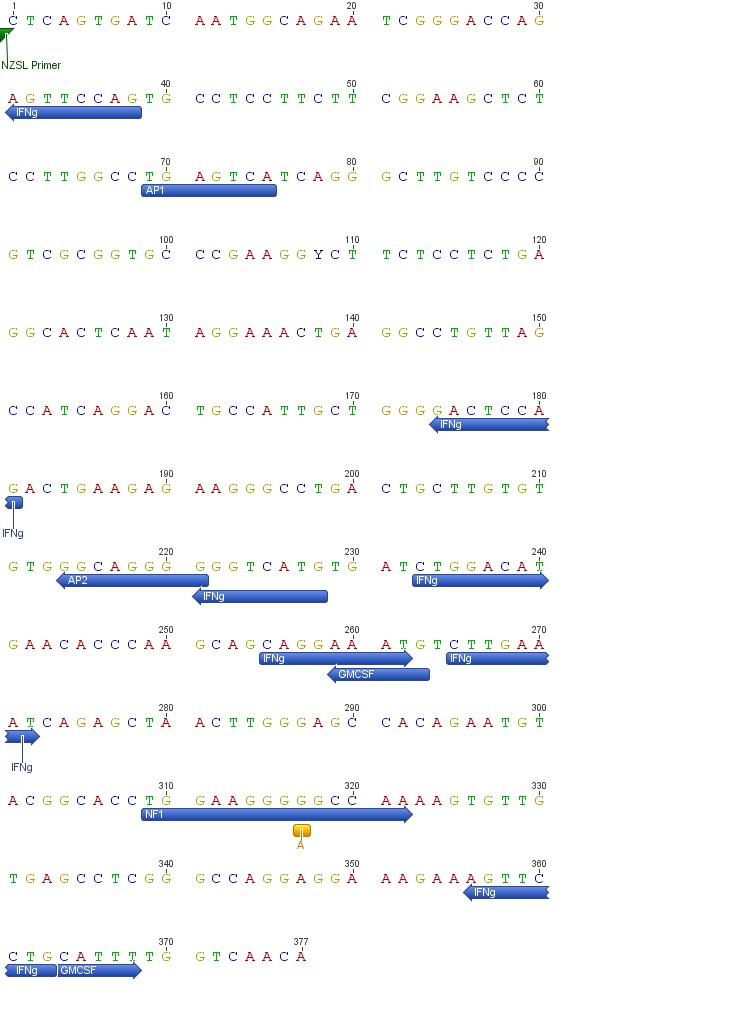


**S2 Fig.** **Transcription factor binding motifs.**

Putative transcription factor binding site motifs (AP1, NF-I, IFN-γ, GMCSF) for NZSL SLC11A1 promoter sequence, with SNP (A - G) indicated in gold. Figure output from Geneious with annotations manually added.
