# Supplementary material for: Examining the Role of Components of Slc11a1 (Nramp1) in the Susceptibility of New Zealand Sea Lions (Phocarctos hookeri) to Disease
Source: PLoS One. 2015 Apr 14;10(4):e0122703. doi: 10.1371/journal.pone.0122703 (PMC4397024; doi:10.1371/journal.pone.0122703)
Supplement: S3 Fig — NZSL sequence for SLC11A1 partial promoter region aligned against canine SLC11A1 promoter region. Green bar represents sequence similarity with white gaps showing divergence. Green arrows indicate where NZSL primers bind, with missing NZSL sequence at these sites due to trimming of the sequence. Red arrow indicates the transcription start site in canine sequence. Pale arrow shows the G-rich stretch in dogs associated with susceptibility to infection. This region is interrupted in the NZSL. (DOCX) [file pone.0122703.s003.docx]

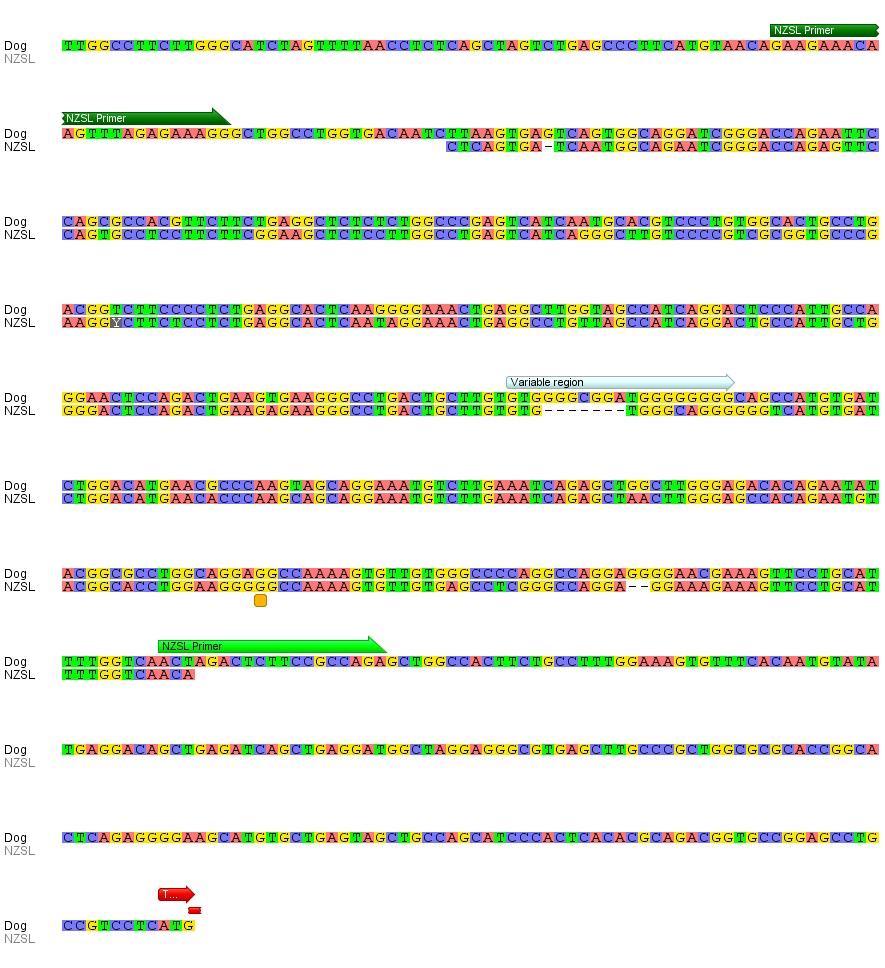


**S3 Fig. Alignment of canine and NZSL partial promoter region.**

NZSL sequence for SLC11A1 partial promoter region aligned against canine SLC11A1 promoter region. Green bar represents sequence similarity with white gaps showing divergence. Green arrows indicate where NZSL primers bind, with missing NZSL sequence at these sites due to trimming of the sequence. Red arrow indicates the transcription start site in canine sequence. Pale arrow shows the G-rich stretch in dogs associated with susceptibility to infection. This region is interrupted in the NZSL.
