# Supplementary material for: Examining the Role of Components of Slc11a1 (Nramp1) in the Susceptibility of New Zealand Sea Lions (Phocarctos hookeri) to Disease
Source: PLoS One. 2015 Apr 14;10(4):e0122703. doi: 10.1371/journal.pone.0122703 (PMC4397024; doi:10.1371/journal.pone.0122703)
Supplement: S4 Fig — Multiple sequence alignment of SLC11A1 sequences from cattle, sheep, pig, human, dog and NZSL. Variable region in canine sequence is shown shaded in blue and promoter SNP is shaded in gold in all species. (DOCX) [file pone.0122703.s004.docx]

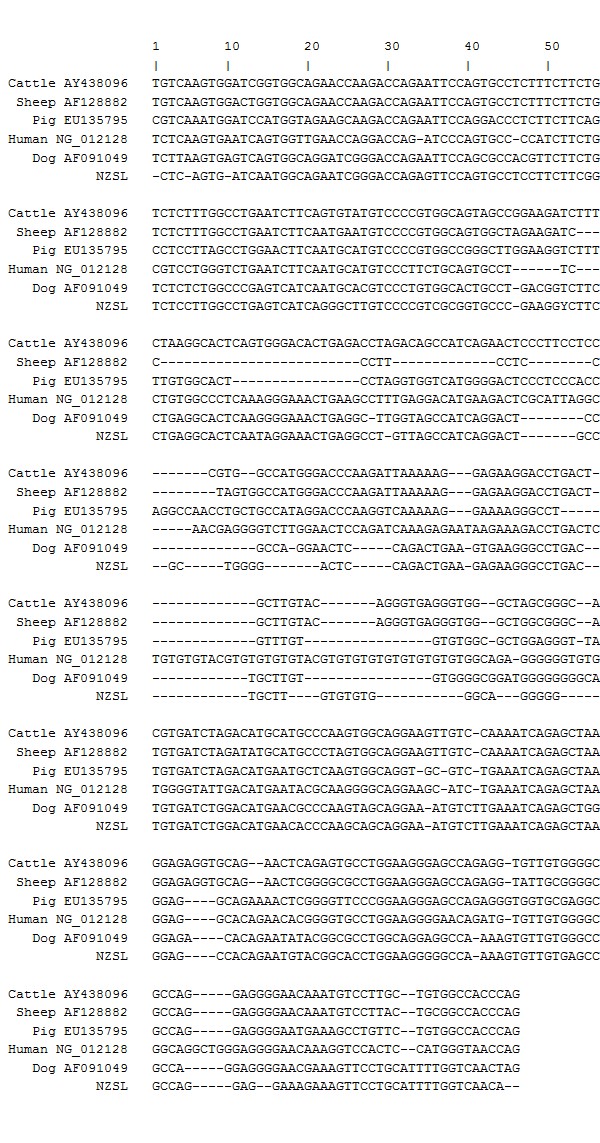


**S4 Fig.** **Mammalian multiple sequence alignment**.

Multiple sequence alignment of SLC11A1 sequences from cattle, sheep, pig, human, dog and NZSL. Variable region in canine sequence is shown shaded in blue and promoter SNP is shaded in gold in all species.
