# Supplementary material for: Examining the Role of Components of Slc11a1 (Nramp1) in the Susceptibility of New Zealand Sea Lions (Phocarctos hookeri) to Disease
Source: PLoS One. 2015 Apr 14;10(4):e0122703. doi: 10.1371/journal.pone.0122703 (PMC4397024; doi:10.1371/journal.pone.0122703)
Supplement: S5 Fig — Haplotype frequencies (%) of combined promoter polymorphism and microsatellite variant, grouped by cause of death. Haplotype (H) numbers refer to haplotype numbers indicated in Table 5. (DOCX) [file pone.0122703.s005.docx]

**S5 Fig.** **Haplotype frequencies.**

Haplotype frequencies (%) of combined promoter polymorphism and microsatellite variant, grouped by cause of death. Haplotype (H) numbers refer to haplotype numbers indicated in Table 5.
